# Analysis of Blocking Rate and Bandwidth Usage of Mobile IPTV Services in Wireless Cellular Networks

**DOI:** 10.1155/2014/215710

**Published:** 2014-10-14

**Authors:** Mingfu Li

**Affiliations:** Department of Electrical Engineering, School of Electrical and Computer Engineering, College of Engineering, Chang Gung University, 259 Wen-Hwa 1st Road, Kwei-Shan, Tao-Yuan 33302, Taiwan

## Abstract

Mobile IPTV services over wireless cellular networks become more and more popular, owing to the significant growth in access bandwidth of wireless cellular networks such as 3G/4G and WiMAX. However, the spectrum resources of wireless cellular networks is rare. How to enhance the spectral efficiency of mobile networks becomes an important issue. Unicast, broadcast, and multicast are the most important transport schemes for offering mobile IPTV services over wireless cellular networks. Therefore, bandwidth usages and blocking rates of unicast, broadcast, and multicast IPTV services were analyzed and compared in this paper. Simulations were also conducted to validate the analytical results. Numerical results demonstrate that the presented analysis is correct, and multicast scheme achieves the best bandwidth usage and blocking rate performance, relative to the other two schemes.

## 1. Introduction

Internet protocol television (IPTV) services have been becoming increasingly popular among telecommunication companies [1, 2]. Recently, due to the significant growth in the amount of mobile devices, access bandwidth over wireless networks, and advanced video coding technologies [3–6], mobile IPTV [7–10] is becoming more feasible over wireless cellular networks. Several works [4–11] have focused on the architecture and standardization for providing mobile IPTV services.

Three basic transport technologies including unicast, broadcast, and multicast can be adopted for delivering mobile IPTV streams. Unicast technology provides one-to-one connection between the base station and the mobile user. Whenever a mobile user requests to watch a TV channel, the base station must establish a dedicated connection for this mobile user if his request is accepted. Even though two mobile users request to watch the same TV channel program, two independent connections must be established, yielding unnecessary bandwidth consumption. To overcome this shortcoming, the broadcast technology [12, 13] can be employed. For broadcast scheme, the base station continuously broadcasts IPTV streams of all channels even though no users watch these channels. In addition, each mobile user can freely switch to the interested TV channel and watch it without sending any request. Thus, several mobile users can watch the same channel program without consuming additional bandwidth resources. However, the drawback of broadcast technology is that the bandwidth resources may be wasted if no users watch the channel programs. As to multicast scheme [12–15], each mobile user must request to join or leave a multicast TV channel group using the Internet group management protocol (IGMP) [16]. If the requested TV channel is in service, the mobile user just joins this channel group directly; otherwise, whether the request is accepted or not depends on the remaining bandwidth resources. Moreover, in multicast scheme, whenever the last viewer of a certain TV channel group leaves the system, the base station immediately stops multicasting this TV channel program and releases the occupied bandwidth resources. Therefore, from the aspect of resource allocation, the major difference between broadcast and multicast schemes is that in multicast the base station needs to allocate bandwidth resources for streaming a channel program only when at least one user watches this channel.

The performance superiority of the transport schemes mentioned above is related to traffic conditions of networks. Therefore, to optimize the resource usage of wireless cellular networks, a hybrid unicast-broadcast delivery scheme was proposed and discussed in [17]. However, how many broadcast channels must be allocated in an optimal hybrid unicast-broadcast delivery scheme remains unsolved. To resolve this issue, the performance of unicast and broadcast services must be analyzed. Related performance analysis on the unicast, broadcast, and/or multicast schemes can be found in [18–22]. The work in [18] aims to determine the optimal number of TV channels served through standard and switched broadcast technologies, so that the access network installation cost can be minimized. The authors in [18] determined the number of simultaneously watched channels, taking content popularity and user behavior into account. But their analysis did not consider the arrival process of TV channel requests and the watching time of each user. Obviously, the contribution of their analysis is limited. The paper [19] presented a wireless switched digital video (WSDV) scheme to increase wireless capacity for mobile TV services. The authors in [19, 20] also developed analytical models to evaluate the TV channel capacity requirement in wireless networks. Similarly, the request arrival process and the watching time of a user were not considered in [19, 20]. Another study [21] takes the request arrival process and the watching time of each user into consideration. In [21], the performance of multicast and broadcast services (MBS) over WiMAX systems was analyzed based on the Poisson arrival process and exponential watching time assumptions. However, their analysis is only an approximation and not verified by simulations. As to the work in [22], the multicast gain of using multicasting to offer mobile IPTV services was defined and evaluated.

The contributions of this work are summarized as follows. Firstly, the user request arrival process, the channel watching time, the channel popularity, the number of available channels, and the limited time-frequency resources are simultaneously considered in the analysis. Secondly, an exact queuing analysis method is introduced to derive blocking rates and bandwidth usages of unicast, broadcast, and multicast IPTV services over wireless cellular networks. Thirdly, simulations are also conducted for validating the correctness of the analysis. Finally, network providers can use the analytical results to design their networks to operate in a more efficient and cost-effective manner.

The rest of this paper is organized as follows.  Section 2 describes the system model.  Section 3 analyzes the performance of unicast and broadcast IPTV services over wireless cellular networks.  Section 4 analyzes the blocking rate and the bandwidth usage of multicast IPTV services. Numerical results and performance comparisons are presented in Section 5. Finally, the concluding remarks are made in Section 6.

## 2. System Model

In addition to traditional voice and data services, mobile IPTV services must also be supported in modern mobile networks. Effective performance analysis for mobile networks that simultaneously support voice, data, and IPTV services is still intractable now. However, since mobile IPTV services require extra large bandwidth compared with voice and data services, the performance of mobile networks is significantly dominated by mobile IPTV traffic. Thus, to make the performance analysis problem tractable, in the first stage a simpler mobile network system in which the dedicated time-frequency resources for voice/data traffic and mobile IPTV traffic are completely separated is assumed. That is, in this paper the voice and data traffic are ignored and only the performance of IPTV services is analyzed.

To understand the effects of mobile IPTV services on the performance of mobile networks, the following queuing model is presented and analyzed. The considered queuing system in this paper is limited to within a cell of the mobile network since the network operation of each cell is similar. The terminology* bandwidth resources* is used to represent the time-frequency resources in the wireless cellular network hereafter. The amount of bandwidth resources consumed by each TV channel, namely, the bandwidth usage per TV channel, is assumed to be the same and equals one unit of bandwidth resources. The total amount of bandwidth resources reserved for mobile IPTV services in each cell is assumed to be *K*. The number of available TV channels provided by the service/network provider is assumed to be *N*. The arrival process of TV requests is presumed to follow the Poisson process with arrival rate *λ*. At each TV request arrival, a TV channel is randomly selected according to the channel popularity or hit rate. The hit rate (or popularity) of CH (channel) *i* is denoted by *h*_*i*_, where ∑_*i*=1_^*N*^ *h*_*i*_ = 1. Without loss of generality, *h*_*i*_ ≥ *h*_*j*_ for all *i* < *j* is assumed in this paper. The watching time of each TV user on CH *i* is exponentially distributed with mean 1/*μ*_*i*_.

## 3. Performance Analysis for Broadcast and Unicast IPTV Services

### 3.1. Broadcast IPTV Services

For broadcast IPTV services, each broadcasted TV channel always occupies a unit of bandwidth resources even though no users watch the channel. Since the total amount of bandwidth resources for mobile IPTV services is *K*, only *K* channels out of *N* can be broadcasted simultaneously in a cell. When *N* ≤ *K*, no blocking can occur in the system. If *N* > *K*, it is reasonable to broadcast only the top *K* popular channels among these *N* channels to minimize the blocking rate. Therefore, the other *N* − *K* unpopular channels, CHs *K* + 1, *K* + 2,…, *N*, are not broadcasted. Accordingly, if *N* > *K*, the blocking rate *P*_*b*_ of broadcast IPTV services can be expressed by

(1) $$\begin{array}{c} P_{b}=\overset{N}{\underset{i=K+1}{\sum }}h_{i}. \end{array}$$

In this paper, the blocking rate is defined as the probability that a TV request is rejected upon its arrival owing to the requested TV channel not in service and the system lack of bandwidth resources to start it.

Next, the bandwidth usage *Y*, which is defined as the amount of bandwidth resources consumed by all TV channels, is equal to

(2) $$\begin{array}{c} Y=\min\left\{N,K\right\}. \end{array}$$

### 3.2. Unicast IPTV Services

For unicast IPTV services, each accepted TV request can be served by using an individual connection between the mobile user and the base station. Therefore, the system in a cell can be simply modeled as an *M*/*M*/*K*/*K* queuing system. For the heterogeneous service rate model, the service rate of state *n* depends on the service rate *μ*_*i*_ and the hit rate *h*_*i*_. Given that the system is in state *n* (*n* connections in the system), there are *N* ^*n*^ possible combinations for these *n* unicast connections. Let all these possible combinations be in the set *R*_*n*_ = {(*l*_1_, *l*_2_,…, *l*_*n*_)∣∀ 1 ≤ *l*_1_, *l*_2_,…, *l*_*n*_ ≤ *N*}. Then the average service rate of state *n*, denoted by ${\overset{-}{\mu }}_{n}$, can be written as

(3) $$\begin{array}{c} {\overset{-}{\mu }}_{n}=\underset{\forall (l_{1},\ldots ,l_{n})\in R_{n}}{\sum }\left(\overset{n}{\underset{i=1}{\sum }}\mu_{l_{i}}\right)\overset{n}{\underset{i=1}{\prod }}h_{l_{i}}. \end{array}$$

The operator ∑_∀(*l*_1_,…,*l*_*n*_)∈*R*_*n*__ in (3) is equivalent to ∑_*l*_1_=1_^*N*^∑_*l*_2_=1_^*N*^ ⋯ ∑_*l*_*n*_=1_^*N*^. The term ∏_*i*=1_^*n*^ *h*_*l*_*i*__ in (3) is the probability that these *n* unicast connections are for CHs *l*_1_, *l*_2_,…, and *l*_*n*_. And the term ∑_*i*=1_^*n*^ *μ*_*l*_*i*__ represents the corresponding service rate of state *n*. Let the number of unicast TV connections be *V*. According to the queuing theory [23], the stationary state probability of state *n* in the *M*/*M*/*K*/*K* model can be expressed by

(4) $$\begin{array}{c} \mathrm{Pr}\left\{V=n\right\}=\frac{\prod_{i=1}^{n}{\overset{-}{r}}_{i}}{1+\sum_{k=1}^{K}\prod_{i=1}^{k}{\overset{-}{r}}_{i}},\;\text{for}\;1\leq n\leq K,\\ \mathrm{Pr}\left\{V=0\right\}=\frac{1}{1+\sum_{k=1}^{K}\prod_{i=1}^{k}{\overset{-}{r}}_{i}}, \end{array}$$

where ${\overset{-}{r}}_{i}=\lambda /{\overset{-}{\mu }}_{i}$. Therefore, the blocking rate *P*_*b*_ of unicast IPTV services becomes

(5) $$\begin{array}{c} P_{b}=\mathrm{Pr}\left\{V=K\right\}=\frac{\prod_{i=1}^{K}{\overset{-}{r}}_{i}}{1+\sum_{k=1}^{K}\prod_{i=1}^{k}{\overset{-}{r}}_{i}}. \end{array}$$

Finally, the bandwidth usage *Y* is equal to *V* for the unicast scheme. Therefore, the mean of *Y* can be derived as follows:

(6) $$\begin{array}{c} E\left(Y\right)=E\left(V\right)=\frac{\sum_{n=1}^{K}n\prod_{i=1}^{n}{\overset{-}{r}}_{i}}{1+\sum_{k=1}^{K}\prod_{i=1}^{k}{\overset{-}{r}}_{i}}. \end{array}$$

#### 3.2.1. Homogeneous Service Rates

For the special case of homogeneous service rates *μ*_*i*_ = *μ*, 1 ≤ *i* ≤ *N*, ${\overset{-}{\mu }}_{n}$ in (3) is equal to *nμ*. Thus, the blocking rate *P*_*b*_ in (5) can be rewritten as

(7) $$\begin{array}{c} P_{b}=\frac{r^{K}/K!}{\sum_{i=0}^{K}\left(r^{i}/i!\right)}, \end{array}$$

where *r* = *λ*/*μ*. And the bandwidth usage *E*(*Y*) in (6) can be simplified to

(8) $$\begin{array}{c} E\left(Y\right)=r\left(1-P_{b}\right). \end{array}$$

#### 3.2.2. Uniform Hit Rate Distribution

For the case of uniform hit rate distribution *h*_*i*_ = 1/*N*, 1 ≤ *i* ≤ *N*, the average service rate ${\overset{-}{\mu }}_{n}$ in (3) reduces to *n*∑_*i*=1_^*N*^ *μ*_*i*_/*N*. Therefore, the blocking rate *P*_*b*_ and the bandwidth usage *E*(*Y*) remain the same as those in (7) and (8), respectively, except that *r* = *Nλ*/∑_*i*=1_^*N*^ *μ*_*i*_.

## 4. Performance Analysis for Multicast IPTV Services

### 4.1. Nonblocking Case: *N* ≤ *K*

When *N* ≤ *K*, no blocking can occur in the system. Each TV channel can be simply modeled as an *M*/*M*/*∞* queuing system. Hence, according to [23] the stationary state distribution of *V*_*i*_, the number of viewers of CH *i* is given by

(9) $$\begin{array}{c} \mathrm{Pr}\left\{V_{i}=n\right\}=\frac{{\left(r_{i}h_{i}\right)}^{n}e^{-r_{i}h_{i}}}{n!}, \end{array}$$

where *r*_*i*_ = *λ*/*μ*_*i*_. Let *X*_*i*_ denote the status of CH *i*, where *X*_*i*_ = 1 if CH *i* is in service (at least one viewer watches CH *i*) and *X*_*i*_ = 0, otherwise. Then one has

(10) $$\begin{array}{c} \mathrm{Pr}\left\{X_{i}=0\right\}=\mathrm{Pr}\left\{V_{i}=0\right\}=e^{-r_{i}h_{i}}. \end{array}$$

Thus,

(11) $$\begin{array}{c} \mathrm{Pr}\left\{X_{i}=1\right\}=1-\mathrm{Pr}\left\{X_{i}=0\right\}=1-e^{-r_{i}h_{i}}. \end{array}$$

For multicast scheme, the bandwidth usage *Y* equals the number of multicasted channels; namely, *Y* = ∑_*i*=1_^*N*^ *X*_*i*_. Therefore, one can deduce

(12) E(Y)=∑i=1NE(Xi)=∑i=1NPr⁡⁡{Xi=1}=∑i=1N(1−e−rihi)=N−∑i=1Ne−rihi.

### 4.2. Blocking Case: *N* > *K*

**Figure 1:**
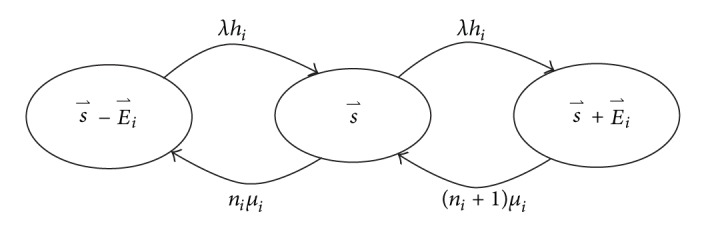
State transition diagram.

Subsequently, let us consider the blocking case *N* > *K*. First, the system state $\vec{s}$ is defined to be the *N*-tuple vector $\vec{s}=(n_{1},n_{2},\ldots ,n_{N})$, where *n*_*i*_ represents the number of viewers of CH *i*. The stationary state probability of $\vec{s}$ is denoted by $p(\vec{s})$. The state transition diagram under the Markovian model is depicted in Figure 1, where ${\vec{E}}_{i}$ = (0,…, 0, *e*_*i*_ = 1, 0,…, 0) is the *N*-tuple vector that all elements are zero except the *i*th element *e*_*i*_ = 1. Channel *i* can be multicasted in a cell only when *n*_*i*_ is greater than zero. Since the total amount of bandwidth resources for mobile IPTV services is *K* and each multicasted channel consumes one unit of bandwidth resources, the nonzero elements in (*n*_1_, *n*_2_,…, *n*_*N*_) cannot be more than *K*.

When the system is in state $\vec{s}$ and the bandwidth usage *Y* satisfies *Y* ≤ *K* − 1, then no user request can be rejected. Hence, according to the state transition diagram in Figure 1, the global balance equation under the condition *Y* ≤ *K* − 1 can be expressed as follows:

(13) p(s→)∑i=1N[niμiI(ni)+λhi] =∑i=1N[λhip(s→−E→i)I(ni)+(ni+1)μip(s→+E→i)],

where *I*(*n*_*i*_) is the indicator function defined by

(14) $$\begin{array}{c} I\left(n_{i}\right)=\{\begin{array}{cc} 1, & \text{if}\;n_{i}>0,\\ 0, & \text{otherwise}. \end{array} \end{array}$$

In (13), the term *n*_*i*_ *μ*_*i*_ must be multiplied by *I*(*n*_*i*_) because when *n*_*i*_ = 0, no viewer is watching CH *i* and the term *n*_*i*_ *μ*_*i*_ must disappear. The term $\lambda h_{i}p(\vec{s}-{\vec{E}}_{i})$ must be multiplied by *I*(*n*_*i*_) because the state $\vec{s}-{\vec{E}}_{i}$ does not exist if *n*_*i*_ = 0.

When the system is in state $\vec{s}$ and the bandwidth usage *Y* equals *K*, then the request for the channel that is not multicasted and no users are watching must be rejected. Hence, the global balance equation under the condition *Y* = *K* becomes

(15) p(s→)∑i=1N[niμi+λhi]I(ni) =∑i=1N[λhip(s→−E→i)+(ni+1)μip(s→+E→i)]I(ni).

In (15), the term *λh*_*i*_ must be multiplied by *I*(*n*_*i*_) because if *n*_*i*_ = 0, the request for CH *i* must be rejected owing to lack of bandwidth resources. The term $\left(n_{i}+1)\mu_{i}p(\vec{s}+{\vec{E}}_{i}\right)$ must be multiplied by *I*(*n*_*i*_) because if *n*_*i*_ = 0, the state $\vec{s}+{\vec{E}}_{i}$ does not exist owing to its bandwidth usage greater than *K*.

It is difficult to solve the stationary state probability $p(\vec{s})$ using the global balance equations (13) and (15). However, the following local balance equation

(16) $$\begin{array}{c} \lambda h_{i}p\left(\vec{s}\right)=\left(n_{i}+1\right)\mu_{i}p\left(\vec{s}+{\vec{E}}_{i}\right) \end{array}$$

can satisfy both global balance equations (13) and (15). The reason is explained as follows. From (16), one can deduce the following equations:

(17) $$\begin{array}{c} \overset{N}{\underset{i=1}{\sum }}\lambda h_{i}p\left(\vec{s}\right)=\overset{N}{\underset{i=1}{\sum }}\left(n_{i}+1\right)\mu_{i}p\left(\vec{s}+{\vec{E}}_{i}\right), \end{array}$$

(18) $$\begin{array}{c} \overset{N}{\underset{i=1}{\sum }}\lambda h_{i}p\left(\vec{s}-{\vec{E}}_{i}\right)I\left(n_{i}\right)=\overset{N}{\underset{i=1}{\sum }}n_{i}\mu_{i}p\left(\vec{s}\right)I\left(n_{i}\right), \end{array}$$

(19) $$\begin{array}{c} \overset{N}{\underset{i=1}{\sum }}\lambda h_{i}p\left(\vec{s}\right)I\left(n_{i}\right)=\overset{N}{\underset{i=1}{\sum }}\left(n_{i}+1\right)\mu_{i}p\left(\vec{s}+{\vec{E}}_{i}\right)I\left(n_{i}\right). \end{array}$$

Obviously, (13) can be derived by combining (17) and (18). Similarly, combining (18) and (19), (15) is obtained.

Accordingly, the stationary state probability $p(\vec{s})$ can be solved using the local balance equation (16). Let *A*_*k*_ denote the set of all vectors $\vec{s}$ with exactly *k* elements in (*n*_1_, *n*_2_,…, *n*_*N*_) being nonzero, where 0 ≤ *k* ≤ *K*. That is,

(20) Ak≡{(n1,n2,…,nN) ∣ exactly  k  elements       in  (n1,n2,…,nN)  are  nonzero}.

Then the sample space of all states, *S*, can be expressed by

(21) $$\begin{array}{c} S=\overset{K}{\underset{k=0}{⋃}}A_{k}. \end{array}$$

Besides, *C*_*k*_ is defined to be the set of all possible combinations of choosing *k* different integers from 1 to *N*.

For all states $\vec{s}=(n_{1},\ldots ,n_{i},\ldots ,n_{N})\in S$ and *n*_*i*_ > 0, the local balance equation in (16) can be rewritten as follows:

(22) λhi·p(n1,…,ni−1,ni−1,ni+1,…,nN) =niμi·p(n1,…,ni−1,ni,ni+1,…,nN).

Then one can iteratively derive the following result

(23) p(n1,…,ni−1,ni,ni+1,…,nN) =(rihi)nini!·p(n1,…,ni−1,0,ni+1,…,nN).

Subsequently, taking the summation of both sides in (23) yields

(24) ∑ni=1∞p(n1,…,ni−1,ni,ni+1,…,nN) =(erihi−1)·p(n1,…,ni−1,0,ni+1,…,nN).

According to (24), for any states $\vec{s}\in A_{1}$, one has

(25) ∑s→∈A1p(s→)=∑i=1N ∑ni=1∞p(0,…,0,ni,0,…,0)=∑i=1N(erihi−1)·p(0→),

where $p(\vec{0})\equiv p(0,\ldots ,0)$. Similarly, for any states $\vec{s}\in A_{2}$, one can obtain

(26) ∑s→∈A2p(s→) =∑∀(i,j)∈C2 ‍∑nj=1∞ ‍∑ni=1∞p(0,…,0,ni,0,…,0,nj,0,…,0) =∑∀(i,j)∈C2 ‍∑nj=1∞ ‍∑ni=1∞(rihi)nini!·p(0,…,0,nj,0,…,0) =∑∀(i,j)∈C2(erihi−1)∑nj=1∞(rjhj)njnj!·p(0→) =∑∀(i,j)∈C2(erihi−1)(erjhj−1)·p(0→).

Iteratively, for any states $\vec{s}\in A_{k}$, the following result can be derived:

(27) $$\begin{array}{c} \underset{\vec{s}\in A_{k}}{\sum }p\left(\vec{s}\right)=\underset{\forall (l_{1},\ldots ,l_{k})\in C_{k}}{\sum }\;‍\overset{k}{\underset{j=1}{\prod }}\left(e^{r_{l_{j}}h_{l_{j}}}-1\right)\cdot p\left(\vec{0}\right). \end{array}$$

The operator ∑_∀(*l*_1_,…,*l*_*k*_)∈*C*_*k*__ in (27) is equivalent to ∑_*l*_1_=1_^*N*^∑_*l*_2_=*l*_1_+1_^*N*^ ⋯ ∑_*l*_*k*_=*l*_*k*−1_+1_^*N*^.

Since the sum of all stationary state probabilities must be equal to 1, the following equation must hold:

(28) p(0→)+∑k=1K ‍∑s→∈Akp(s→) =p(0→){1+∑k=1K ‍∑∀(l1,…,lk)∈Ck ‍∏j=1k(erljhlj−1)}=1.

Thus,

(29) $$\begin{array}{c} p\left(\vec{0}\right)=\frac{1}{1+\sum_{k=1}^{K}\sum_{\forall (l_{1},\ldots ,l_{k})\in C_{k}}^{}\prod_{j=1}^{k}\left(e^{r_{l_{j}}h_{l_{j}}}-1\right)}. \end{array}$$

Applying the similar procedure in (23) to other elements *n*_*j*_'s, the stationary state probability $p(\vec{s})$ can be expressed by

(30) $$\begin{array}{c} p\left(\vec{s}\right)=p\left(\vec{0}\right)\overset{N}{\underset{i=1}{\prod }}\frac{{\left(r_{i}h_{i}\right)}^{n_{i}}}{n_{i}!}. \end{array}$$

The blocking rate *P*_*b*_ can be computed according to the following equation:

(31) $$\begin{array}{c} P_{b}=\underset{\vec{s}\in A_{K}}{\sum }\mathrm{Pr}\left\{\text{request}\;\text{rejected}\;|\;\vec{s}\right\}p\left(\vec{s}\right), \end{array}$$

where $\mathrm{Pr}\left\{\text{request}\;\text{rejected}|\vec{s}\right\}$ is the conditional probability that a request is rejected upon its arrival, given that the system is in the state $\vec{s}$. Notably, a request can be rejected only when the system is in the state with bandwidth usage *Y* = *K*; that is, $\vec{s}\in A_{K}$, and the requested CH *i* is not multicasted (*n*_*i*_ = 0). Assume the system is in the state $\vec{s}$ with nonzero elements *n*_*l*_1__,…, *n*_*l*_*K*__; then only CHs *l*_1_,…, *l*_*K*_ are multicasted. Hence, only the request for CH *l*_*i*_, 1 ≤ *i* ≤ *K*, can be accepted, with the total acceptance probability ∑_*j*=1_^*K*^ *h*_*l*_*j*__. Accordingly, the conditional probability that a request is rejected upon its arrival, given that the system is in the state $\vec{s}\in A_{K}$, is given by

(32) $$\begin{array}{c} \mathrm{Pr}\left\{\text{request}\;\text{rejected}\;|\;\vec{s}\right\}=1-\overset{K}{\underset{j=1}{\sum }}h_{l_{j}}. \end{array}$$

Based on (27), (29), (31), and (32), the blocking rate can be expressed by

(33) $$\begin{array}{c} P_{b}=\frac{\sum_{\forall (l_{1},\ldots ,l_{K})\in C_{K}}^{}\left(1-\sum_{j=1}^{K}h_{l_{j}}\right)\prod_{j=1}^{K}\left(e^{r_{l_{j}}h_{l_{j}}}-1\right)}{1+\sum_{k=1}^{K}\sum_{\forall (l_{1},\ldots ,l_{k})\in C_{k}}^{}\prod_{j=1}^{k}\left(e^{r_{l_{j}}h_{l_{j}}}-1\right)}. \end{array}$$

As to the average bandwidth usage, *E*(*Y*), it can be obtained as follows:

(34) $$\begin{array}{c} E\left(Y\right)=\frac{\sum_{k=1}^{K}k\sum_{\forall (l_{1},\ldots ,l_{k})\in C_{k}}^{}\prod_{j=1}^{k}\left(e^{r_{l_{j}}h_{l_{j}}}-1\right)}{1+\sum_{k=1}^{K}\sum_{\forall (l_{1},\ldots ,l_{k})\in C_{k}}^{}\prod_{j=1}^{k}\left(e^{r_{l_{j}}h_{l_{j}}}-1\right)}. \end{array}$$

## 5. Numerical Results

In this section, blocking rates and bandwidth usages of unicast, broadcast, and multicast IPTV services based on the analytical results given in previous sections are evaluated. Simulation results are also given for verification. In addition, the effects of hit rate distribution on the performance are investigated as well. Several hit rate distributions, such as uniform and Zipf-like [2, 18–21] distributions, are considered. In this section, only the numerical results for the blocking case *N* > *K* are presented. In all examples, the parameter *N* is set to 25 and *K* equals 20.

**Figure 2:**
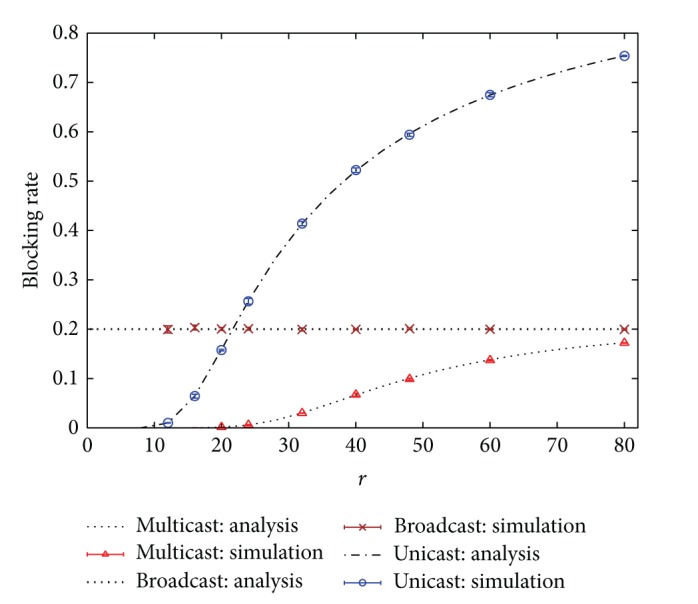
Blocking rates of unicast, broadcast, and multicast IPTV services. (Hit rate distribution: uniform.)

**Figure 3:**
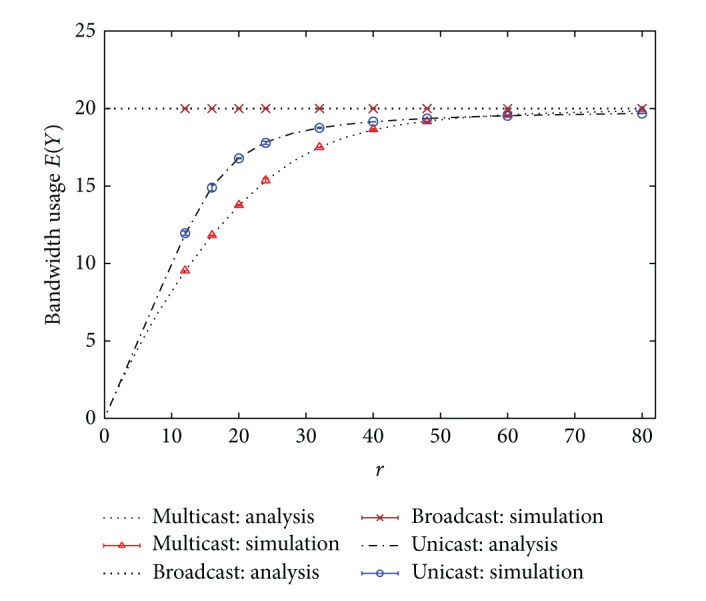
Bandwidth usages of unicast, broadcast, and multicast IPTV services. (Hit rate distribution: uniform.)

In the first example, homogeneous service rates are considered, that is, *μ*_*i*_ = *μ* for all *i*. The hit rate is assumed to have the uniform distribution. Figures 2 and 3 show the blocking rate and the bandwidth usage performance, respectively, under different values of *r* = *λ*/*μ*. Simulation results with 99% confidence intervals are also given in the figures. Simulation results in Figures 2 and 3 demonstrate that the analytical results are extremely accurate. Moreover, the performance of multicast scheme is the best, relative to the other two schemes. The blocking rate of unicast scheme becomes unacceptably high when *r* is large, as shown in Figure 2. However, the performance of broadcast scheme remains unchanged under different values of *r*. It reveals that the broadcast scheme cannot adapt to different traffic conditions so that its bandwidth efficiency is worse, as displayed in Figure 3. Notably, one cannot judge the superiority of a scheme solely based on the bandwidth usage, since a smaller bandwidth usage may result from a higher blocking rate. For example, in Figure 3 the bandwidth usage of unicast scheme is smaller than that of broadcast scheme, even slightly smaller than that of multicast scheme at heavy load. This is because at heavy load lots of users are blocked from entering the system in unicast scheme so that its bandwidth usage is reduced. However, one can observe that no matter under the light or heavy load, both the blocking rate and the bandwidth usage of multicast scheme are almost better than those of the other two schemes. Hence, multicast scheme is the best candidate for offering mobile IPTV services over wireless cellular networks, from the aspects of blocking rate and bandwidth usage.

**Figure 4:**
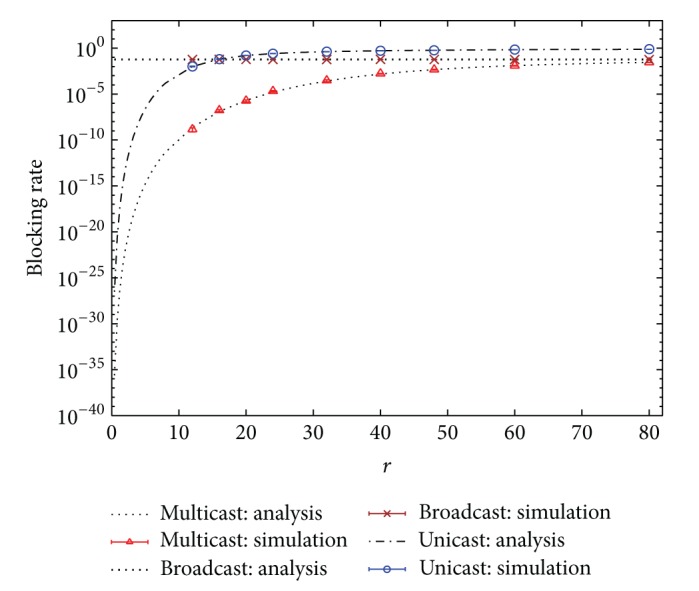
Blocking rates of unicast, broadcast, and multicast IPTV services. (Hit rate distribution: Zipf-like distribution with *a* = 1.)

**Figure 5:**
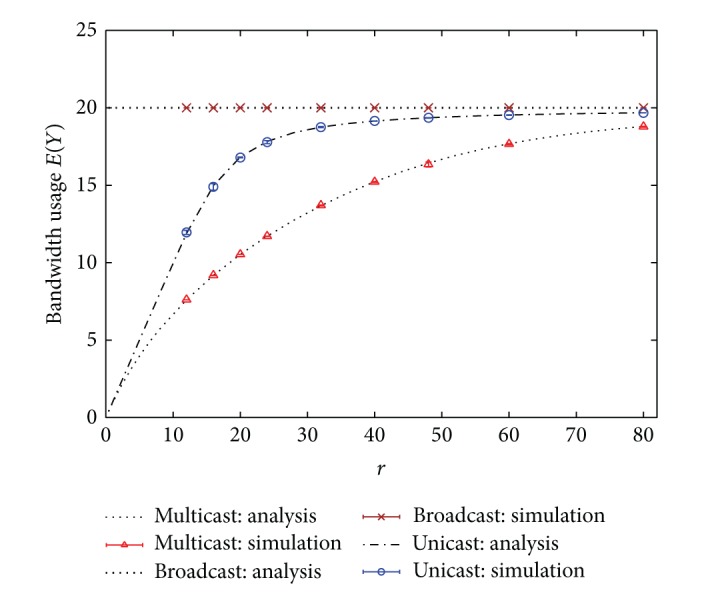
Bandwidth usages of unicast, broadcast, and multicast IPTV services. (Hit rate distribution: Zipf-like distribution with *a* = 1.)

In the second example, the hit rate distribution is assumed to follow the Zipf-like distribution. That is, the hit rate is distributed according to the formula

(35) $$\begin{array}{c} h_{i}=\frac{i^{-a}}{\sum_{n=1}^{N}n^{-a}}, \end{array}$$

where *a* is the exponent parameter. The other system parameters of the second example are the same as those in the first one except that the Zipf-like distribution with parameter *a* = 1 is adopted as the hit rate distribution. Figures 4 and 5 indicate the blocking rate and the bandwidth usage performance, respectively. Simulation results with 99% confidence intervals are also included. Similar conclusions can be made as in the first example. Additionally, compared with Figure 2, the blocking rates of multicast and broadcast schemes in Figure 4 are significantly reduced. The bandwidth usage of multicast scheme in Figure 5 is also reduced, relative to Figure 3. As to the performance of unicast scheme, it is irrelevant to the hit rate distribution according to (7) and (8). Furthermore, in Figure 4, when *r* increases larger and larger, the blocking rate of unicast scheme approaches closer and closer 1 because the bandwidth resources has been exhausted after the number of TV users reaches *K*, while for multicast scheme the combination of multicasted channels is almost similar to that of broadcast scheme as the number of TV users becomes more and more. Therefore, the blocking rate of multicast scheme approaches that of broadcast scheme when *r* is large, as displayed in Figure 4.

**Figure 6:**
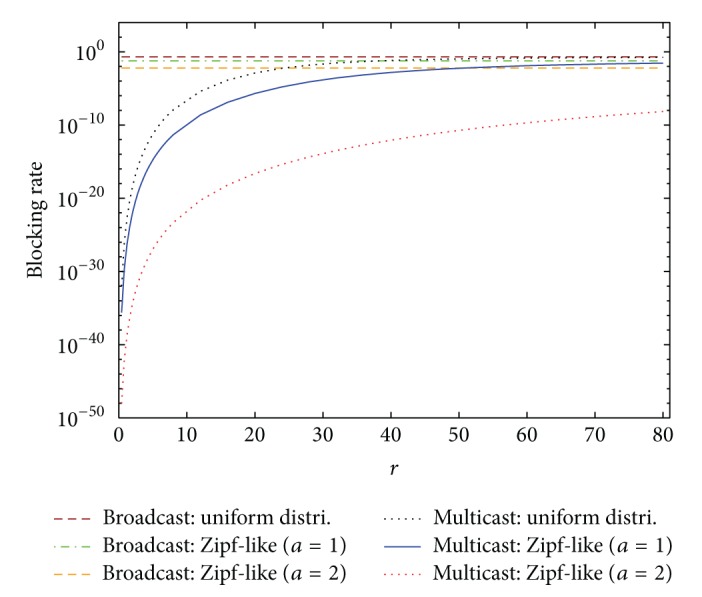
Blocking rates of broadcast and multicast IPTV services under different hit rate distributions.

**Figure 7:**
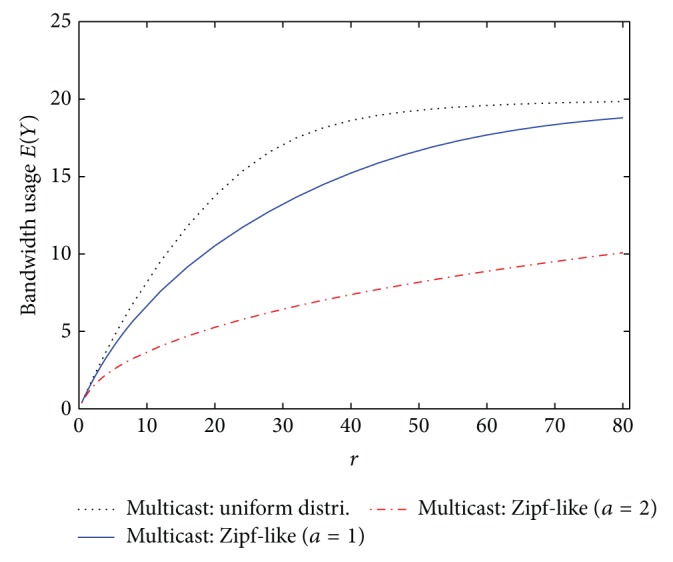
Bandwidth usage of multicast IPTV services under different hit rate distributions.

Based on the previous two examples, the performance of broadcast and multicast schemes is related to the hit rate distribution. Therefore, the effects of hit rate distribution on the performance of IPTV services are studied herein. The system parameters are set the same as those in the first example except for the hit rate distribution.  Figure 6 displays the blocking rate performance of broadcast and multicast schemes under various hit rate distributions. Since the bandwidth usage *E*(*Y*) of broadcast scheme always equals min⁡{*N*, *K*}, which is irrelevant to the hit rate *h*_*i*_, Figure 7 only shows the bandwidth usage of multicast scheme. Compared with the uniform distribution, the hit rates are more skewed in the Zipf-like distribution [2, 21]. And the larger the parameter *a* in the Zipf-like distribution is, the more skewed the hit rates will be. When some TV channels become much more popular than the others, namely, with more skewed hit rates, almost all the mobile users watch these popular TV channels. Hence, in multicast scheme the number of multicasted channels reduces, yielding the result that the bandwidth usage *E*(*Y*) decreases and more bandwidth resources becomes available. Accordingly, for multicast scheme, the blocking rate and the bandwidth usage under the uniform hit rate distribution are worse, relative to the Zipf-like distribution with parameter *a* = 1 or *a* = 2, as shown in Figures 6 and 7. As to broadcast scheme, the blocking rate is equal to the total hit rate of the top *N* − *K* unpopular channels. When the hit rates become more skewed, the hit rates of popular channels increase while the hit rates of unpopular channels decrease. Consequently, the blocking rate of broadcast scheme becomes smaller when the hit rates are more skewed.

Finally, the performance of unicast and multicast services under the scenario of heterogeneous service rates is studied. In the following, the service rate *μ*_*i*_ is set to be

(36) $$\begin{array}{c} \mu_{i}=\frac{1}{20-0.5\times i}. \end{array}$$

**Figure 8:**
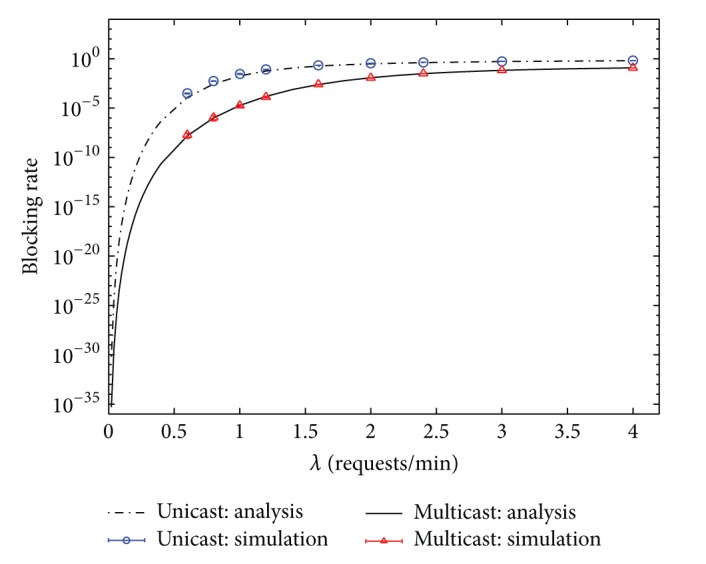
Blocking rates of unicast and multicast IPTV services under the scenario of heterogeneous service rates. (Hit rate distribution: uniform.)

**Figure 9:**
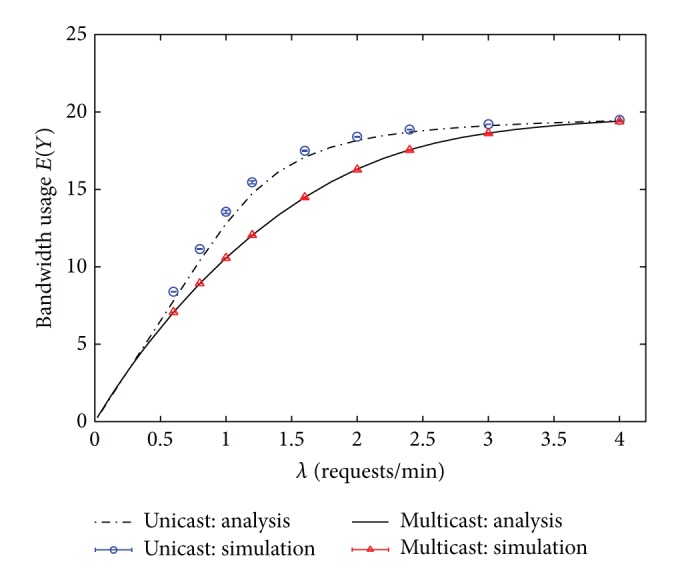
Bandwidth usages of unicast and multicast IPTV services under the scenario of heterogeneous service rates. (Hit rate distribution: uniform.)

The unit of *μ*_*i*_ is request/min. In (36), the average channel watching time 1/*μ*_*i*_ of a popular channel (with a smaller *i*) is assumed to be longer than that of an unpopular one (with a larger *i*). Uniform hit rate distribution is considered here. Other parameters not mentioned here are the same as those in the first example. The blocking rates of unicast and multicast services are displayed in Figure 8, while the bandwidth usages are shown in Figure 9. Since the blocking rate and the bandwidth usage of broadcast IPTV services are irrelevant to the service rates *μ*_*i*_, Figures 8 and 9 do not include the performance of broadcast IPTV services. Simulation results with 99% confidence intervals are given in Figures 8 and 9 for validating the correctness of analytical results. Figures 8 and 9 demonstrate that the analytical results are accurate even under the case of heterogeneous service rates.

## 6. Conclusions

Blocking rates and bandwidth usages of unicast, broadcast, and multicast IPTV services over wireless cellular networks are analyzed in this paper. In our analysis, the arrival process of TV channel requests, channel watching time, and channel popularity are concurrently considered. The analytical results are validated to be correct by simulations. The effects of channel hit rates on the performance of mobile IPTV services are also investigated in the numerical results. Based on the numerical results, multicast scheme is shown to be the best scheme for providing mobile IPTV services over wireless cellular networks from the viewpoints of blocking rate and bandwidth usage. Namely, using multicast scheme to offer mobile IPTV services can minimize the blocking rate and at the same time improve the spectral efficiency of wireless cellular networks. The analytic results in this work can be employed for precise resource allocation and service planning in wireless cellular networks that support mobile IPTV services.

The analytical results derived in this paper are based on the assumption that the bandwidth overheads of unicast, broadcast, and multicast schemes are the same. If the bandwidth overheads of unicast and multicast schemes are different [22], then a hybrid unicast-multicast scheme can be considered. This will be the future work. Additionally, the performance analysis of mobile IPTV services under a more general model that considers the adaptive modulation and coding (AMC) scheme and differentiated services including voice and data traffic will be another future work.
